# Selective Depletion of CREB in Serotonergic Neurons Affects the Upregulation of Brain-Derived Neurotrophic Factor Evoked by Chronic Fluoxetine Treatment

**DOI:** 10.3389/fnins.2018.00637

**Published:** 2018-09-20

**Authors:** Katarzyna Rafa-Zabłocka, Grzegorz Kreiner, Monika Bagińska, Irena Nalepa

**Affiliations:** Department of Brain Biochemistry, Institute of Pharmacology, Polish Academy of Sciences, Kraków, Poland

**Keywords:** BDNF, NTF3, NGF, CREB, CREM, fluoxetine, serotonergic system

## Abstract

Neurotrophic factors are regarded as crucial regulatory components in neuronal plasticity and are postulated to play an important role in depression pathology. The abundant expression of brain-derived neurotrophic factor (BDNF) in various brain structures seems to be of particular interest in this context, as downregulation of BDNF is postulated to be correlated with depression and its upregulation is often observed after chronic treatment with common antidepressants. It is well-known that BDNF expression is regulated by cyclic AMP response element-binding protein (CREB). In our previous study using mice lacking CREB in serotonergic neurons (Creb1^TPH2CreERT2^ mice), we showed that selective CREB ablation in these particular neuronal populations is crucial for drug-resistant phenotypes in the tail suspension test observed after fluoxetine administration in Creb1^TPH2CreERT2^ mice. The aim of this study was to investigate the molecular changes in the expression of neurotrophins in Creb1^TPH2CreERT2^ mice after chronic fluoxetine treatment, restricted to the brain structures implicated in depression pathology with profound serotonergic innervation including the prefrontal cortex (PFC) and hippocampus. Here, we show for the first time that BDNF upregulation observed after fluoxetine in the hippocampus or PFC might be dependent on the transcription factor CREB residing, not within these particular structures targeted by serotonergic projections, but exclusively in serotonergic neurons. This observation may shed new light on the neurotrophic hypothesis of depression, where the effects of BDNF observed after antidepressants in the hippocampus and other brain structures were rather thought to be regulated by CREB residing within the same brain structures. Overall, these results provide further evidence for the pivotal role of CREB in serotonergic neurons in maintaining mechanisms of antidepressant drug action by regulation of BDNF levels.

## Introduction

The majority of the current antidepressant therapies are based on the enhancement of monoaminergic transmission observed directly after drug administration, yet alleviation of depressive symptoms occur several weeks later. The mechanism of molecular changes underlying this phase of adaptation required for antidepressants to become effective remains elusive and is intensively researched. Neurotrophic factors are regarded as crucial regulatory components in neuronal plasticity and are postulated to play an important role in depression pathology (Lee and Kim, 2010). The abundant expression of brain-derived neurotrophic factor (BDNF) in various brain structures seems to be of particular relevance in this context.

It is well-known that BDNF contributes to mechanisms of learning and memory by modulation of synaptic transmission and plasticity (Huang et al., 1999). Patients with depression are often associated with memory impairments, in particular regarding positive events, while memory for negative ones is potentiated (Dillon and Pizzagalli, 2018). According to the cognitive model of depression proposed by Beck, patients with major depressive disorder (MDD) are characterized by impaired cognitive processes, such as attention and memory, experiencing biased processing, rumination with dysfunctional attitudes and negative schemes (Disner et al., 2011). Alterations in BDNF levels may straightforwardly influence activity-dependent plasticity in the hippocampus, therefore having direct impact on memory and emotions in patients with MDD (Phillips, 2017).

In animal models, it has been shown that the expression of BDNF is downregulated by exposure to stress (an important factor contributing to depression) (Smith et al., 1995) and can be restored by antidepressant treatment (Warner-Schmidt and Duman, 2006). These studies were also supported by findings revealing that BDNF is upregulated after chronic treatment with common antidepressants (Russo-Neustadt et al., 1999). Overall, these and many similar observations have prompted the basis of the so-called neurotrophic hypothesis of depression, which presumes that the disease may be related to reduced BDNF levels (particularly in the hippocampus), and thus may be possible to treat with antidepressants that promote neurogenesis enhancement (Duman, 2002).

However, the relationship between antidepressants and neurotrophic factors (mainly BDNF) is complex and structure dependent. Indeed, in humans, it was shown that plasma levels of BDNF are decreased in depression (Cunha et al., 2006) but that downregulation of BDNF in the brain structures was restricted to the hippocampus and prefrontal cortex (PFC), while BDNF levels were increased in the nucleus accumbens and amygdala (Autry and Monteggia, 2012). On the other hand, the effects of antidepressants on BDNF exertion in the hippocampus are rather robust and concomitant (Russo-Neustadt and Chen, 2005), yet they may have opposite effects on other brain structures, i.e., the nucleus accumbens (Berton et al., 2006).

Brain-derived neurotrophic factor encoding gene belongs to the group of genes with a cyclic AMP (cAMP) response element in their promoter region, which is directly regulated by the cAMP response element-binding protein (CREB) cellular transcription factor. Therefore, it is not surprising that many studies have also pointed out an important role for CREB in the mechanisms of antidepressant drug action, although the data on this topic are inconclusive (Nibuya et al., 1996; Dowlatshahi et al., 1998; Rossby et al., 1999; Conti et al., 2002; Yamada et al., 2003; Blendy, 2006; Hisaoka et al., 2008).

Again, despite the variety of data showing that antidepressants upregulate CREB in the hippocampus (Gass and Riva, 2007), mice with hippocampal CREB deletion not only maintained their responsiveness to antidepressants in behavioral tests but also exhibited increased hippocampal neurogenesis observed after antidepressant treatment (Gundersen et al., 2013).

In our previous study, we investigated the role of CREB in the mechanism of antidepressant drug action using newly developed and characterized inducible transgenic mice lacking CREB selectively in serotonergic neurons (Creb1^TPH2CreERT2^ mice). To avoid the well-known compensatory effects of another transcription factor, CREM (McPherson and Lawrence, 2007), which is often neglected by other knock-out studies of CREB function, the animals were maintained in a CREM-deficient background (Creb1^TPH2CreERT2^Crem−/− mice). Although the transgenic mice did not reveal any visible impairments at the basal state, we found that single Creb1^TPH2CreERT2^ mutants resulted in a drug-resistant phenotype in the tail suspension test (TST) after fluoxetine administration and that this effect differed across sex in Creb1^TPH2CreERT2^Crem−/− mice, in that the anxiolytic effect of fluoxetine was restored in male but not female double mutants (Rafa-Zablocka et al., 2017).

The aim of the current study was to investigate the molecular changes in neurotrophin expression in Creb1^TPH2CreERT2^ and Creb1^TPH2CreERT2^Crem−/− mice after chronic fluoxetine treatment, with a focus on the brain structures implicated in depression pathology and with profound innervation by serotonergic projections – the PFC and hippocampus.

## Materials and Methods

### Animals

Selective ablation of CREB in serotonergic neurons (Creb1^TPH2Cre^ mice) was achieved by the Cre/loxP recombination system as described previously (Rafa-Zablocka et al., 2017). Briefly, transgenic mice (C57Bl/6N background) hosting Cre recombinase under the tryptophan hydroxylase 2 (TPH2) promoter (TPH2CreERT2 mice) were crossed with animals harboring the floxed Creb1 gene in a CREM-deficient (Crem−/−) background. The mutation was triggered by application of tamoxifen (2 mg/mouse, 1x daily, five consecutive days; Sigma-Aldrich, United States). Therefore, the resulting transgenic line (Creb1^TPH2CreERT2^Crem−/− mice) possessed a functional deletion of CREB restricted to only serotonergic neurons. Genotyping was performed with a commercially available kit (AccuStart^TM^ II Mouse Genotyping Kit, QuantaBio/VWR) according to the manufacturer’s protocol as described previously (Rafa-Zablocka et al., 2017).

The study was carried out on male and female Creb1^TPH2CreERT2^Crem−/− mice housed with their control (Cre-negative or/and CREM+/+) littermates of the same sex in self-ventilated cages (Allentown, PA, United States) under standard laboratory conditions (12 h light/dark cycle, with food and water *ad libitum*). The study was carried out following the guidance of the Guide for the Care and Use of Laboratory Animals of the National Institutes of Health. All experimental procedures were approved by the Animal Ethical Committee at the Institute of Pharmacology, Polish Academy of Sciences (Permit No. 1125, issued 11/24/2014).

### Drugs and Tissue Collection

**FIGURE 1:**
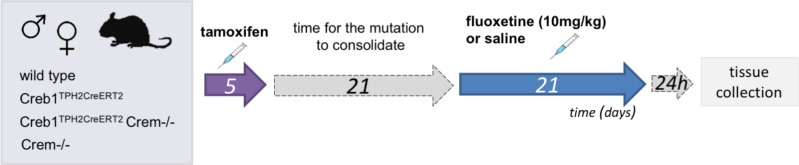
Flowchart summarizing the experimental paradigm.

Three weeks after tamoxifen administration animals of all genotypes: wild-type, Creb1^TPH2CreERT2^, Creb1^TPH2CreERT2^Crem−/−, and Crem−/− were divided into two groups. Control group received saline and treatment group received fluoxetine (10 mg/kg, ip; CarboSynth, United Kingdom) 1x day for 21 consecutive days. Animals were sacrificed by cervical dislocation 24 h after last injection, and tissues were collected (hippocampus and PFC for mRNA/protein assessment, whole brains for immunofluorescence). Experimental scheme is summarized on **Figure 1**.

### Immunofluorescence

The procedure was performed as described previously (Kiryk et al., 2013). Briefly, the brains were removed, fixed overnight in 4% paraformaldehyde (PFA), dehydrated, embedded in paraffin, and coronally sectioned (7 μm) on a rotary microtome (Leica, RM45). Select sections from the corresponding region of the PFC and hippocampus (HIP) were incubated overnight at 4°C with primary anti-BDNF (1:100, Abcam, United Kingdom) antibody and visualized with fluorescent anti-rabbit Alexa-488 secondary antibody (Invitrogen, United States). Stained sections were acquired and analyzed under a fluorescence microscope (Nikon Eclipse50i, Japan) equipped with a camera and NIS Elements software.

### Real-Time PCR

The procedure was performed as described previously (Diaz-Ruiz et al., 2008). Briefly, after dissection, the brain structures were preserved in RNAlater (Ambion, United States), RNA was extracted using an RNeasy Mini kit (Qiagen, United States), its quality was verified on an Agilent 2100 Bioanalyzer, and the quantity was spectrophotometrically determined. Reverse transcription was performed on 1000 ng of total RNA from each sample using MultiScribe Reverse Transcriptase (Applied Biosystems, United States). TaqMan qPCR was performed on 50 ng reverse-transcribed cDNA in a final volume of 20 μl using the Quant Studio Platform (Life Technologies, United States) following the manufacturer’s protocol. The following predesigned TaqMan gene expression assays were used: Creb1 (Mm00501607_m1), Ntrk2 (Mm00435422_m1), Bdnf (Mm04230607_s1), Ntrk3 (Mm00456222_m1), Ntf3 (Mm00435413_s1), and Ngf (Mm00443039_m1). Hypoxanthine-phosphoribosyltransferase (Hprt1, Mm03024075_m1) was chosen as the housekeeping gene. The results were calculated as the fold change in expression compared to that in the control mice (wild-type littermates) using the ΔΔCt method.

### Western Blot

For protein isolation, each sample was homogenized in RIPA buffer (Sigma, United States) containing a protease inhibitor cocktail (Sigma, United States) and phosphatase inhibitors (Thermo Fisher, United States), incubated for 2 h at 4°C and centrifuged at 18000 × *g*. The protein concentration was assessed by a BCA protein assay kit (Sigma, United States). Samples containing 15 μg protein each were run on a polyacrylamide gel (BioRad, United States) and transferred to nitrocellulose membranes. The membranes were blocked in 5% (w/v) non-fat dry milk in TBST, and the blots were incubated overnight with primary antibodies (dilution: 1:1000) against the following proteins: BDNF (ab108319, Abcam, United Kingdom), CREB (ab32515, Abcam, United Kingdom), phospho-CREB (06-519, Millipore, United States), NGF (sc-365944, Santa Cruz, United States) and NTF3 (PA5-14861, Thermo Fisher, United States). GAPDH (1:5000, MAB374, Millipore, United States) was used as the loading control. After incubation with the proper secondary antibody linked to horseradish peroxidase (dilution 1:5000, anti-mouse PI-2000 and anti-rabbit PI-1000, VECTOR Laboratories, United States), the signal was developed and visualized by WesternBright Quantum (Advansta, United States) HRP substrate with the help of the PXi 4 (Syngene, United Kingdom) imaging system. Densitometric analysis was performed using Multi-Gauge v.3.0 (Fujifilm, Japan) software.

### Statistical Analysis

Data were analyzed using GraphPad Prism 7.0 software (GraphPad, United States). All comparisons were performed using two-way analysis of variance (ANOVA) followed by Fisher’s least significant difference *post hoc* test. Changes with *p*-values lower than 0.05 were considered significant.

## Results

### Selective Ablation of CREB in Serotonergic Neurons Does Not Influence the Expression Levels of mRNA Encoding for CREB and BDNF After Fluoxetine Treatment

To determine whether the mutation impacted the effects of fluoxetine administration on neurotrophic factors, we screened the mRNA expression of Creb1, Bdnf, Ntf3, and Ngf as well as of the receptors of Bdnf and Ntf3, Ntrk2 (TrkB), and Ntrk3 (TrkC), respectively. The experiments were performed on untreated and treated wild-type (w/t) C57BL/6N mice, Creb1^TPH2CreERT2^ mice (single mutants lacking CREB only) and Creb1^TPH2CreERT2^Crem−/− mice (double mutants lacking CREB and CREM). We narrowed the analysis to the two structures widely implicated in depression pathophysiology and the effects of antidepressant drugs – the hippocampus and PFC (Dusi et al., 2015). Taking into account the growing awareness and importance of gender differences in neuropsychiatric disorders and reactiveness to antidepressant treatment (Kreiner et al., 2013), particularly those targeting the serotonergic system in both clinical and experimental studies (Martenyi et al., 2001; Jones and Lucki, 2005; Keers and Aitchison, 2010; Chmielarz et al., 2013), male and female cohorts were used in all experiments.

**FIGURE 2:**
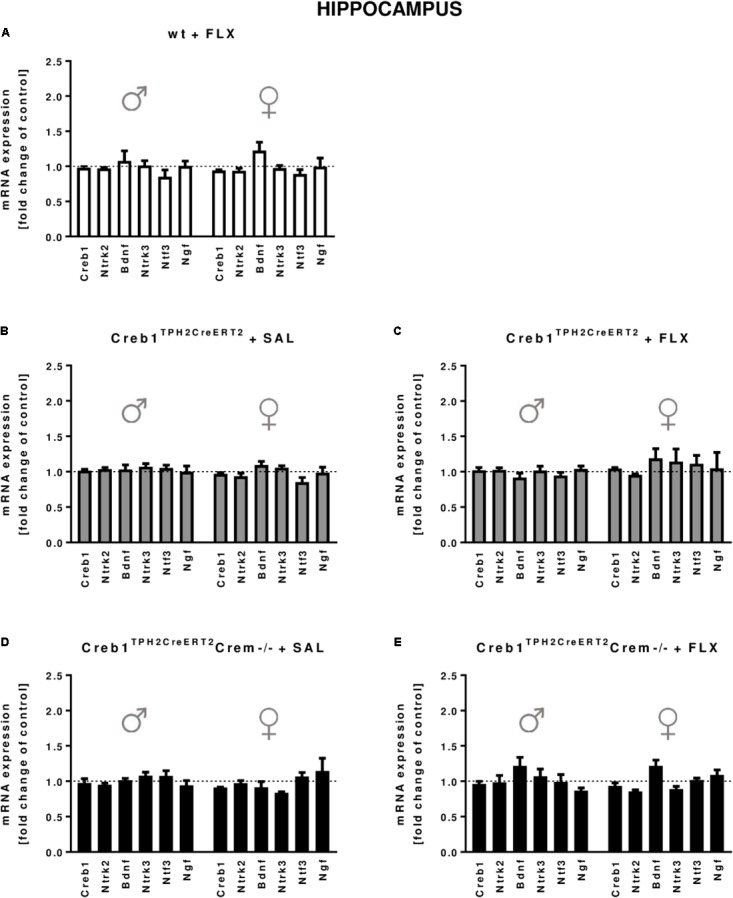
mRNA expression of genes encoding for Creb1, neurotrophins (Bdnf, Ngf, and Ntf3) and their receptors (Ntrk2, Ntrk3) in the hippocampus of saline- and fluoxetine-treated wild-type, Creb1^TPH2CreERT2^, and Creb1^TPH2CreERT2^Crem–/– mice. Neither genotype nor fluoxetine influenced the mRNA expression of Creb1, Bdnf, Ngf, Ntf3, Ntrk2, and Ntrk3. Bars represent fold changes in the mRNA expression of Creb1, Bdnf, Ngf, Ntf3, Ntrk2, and Ntrk3 vs. that in wild-type non-treated animals (dot line) in the hippocampus of **(A)** wild-type fluoxetine-treated, **(B,C)** Creb1^TPH2CreERT2^ saline- and fluoxetine-treated, and **(D,E)** Creb1^TPH2CreERT^Crem–/– saline- and fluoxetine-treated mice. All graphs show data from males (left) and females (right). W/t, wild-type; FLX, fluoxetine. Data are presented as the mean ± SEM.

**FIGURE 3:**
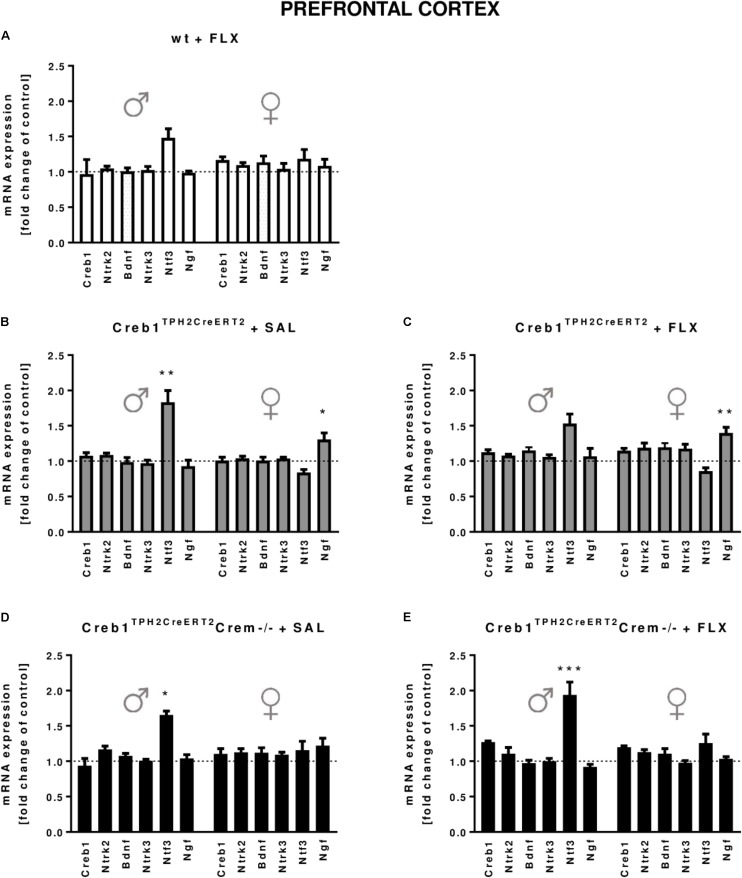
mRNA expression of genes encoding for Creb1, neurotrophins (Bdnf, Ngf, and Ntf3) and their receptors (Ntrk2, Ntrk3) in the prefrontal cortexes (PFCs) of saline- and fluoxetine-treated wild-type, Creb1^TPH2CreERT2^, and Creb1^TPH2CreERT2^Crem–/– mice. In the PFC of male Creb1^TPH2CreERT2^ (saline-treated mice) and Creb1^TPH2CreERT2^Crem–/– mutants (saline- and fluoxetine-treated mice), increased NTF3 mRNA levels were observed. However, Creb1^TPH2CreERT2^ females after both saline and fluoxetine treatment show increased Ngf mRNA expression levels. Bars represent fold changes in the mRNA expression of Creb1, Bdnf, Ngf, Ntf3, Ntrk2, and Ntrk3 vs. that in wild-type non-treated animals (dot line) in the PFC of **(A)** wild-type fluoxetine-treated, **(B,C)** Creb1^TPH2CreERT2^ saline- and fluoxetine-treated, and **(D,E)** Creb1^TPH2CreERT^Crem–/– saline- and fluoxetine-treated mice. All graphs represent data from males (left) and females (right). Data are presented as the mean ± SEM. ^∗^*p* < 0.05, ^∗∗^*p* < 0.01, ^∗∗∗^*p* < 0.001 vs. saline-treated wild-type mice of the same sex. W/t, wild-type; FLX, fluoxetine.

In the hippocampus, we did not observe any changes in the expression of the transcripts investigated, with regard to the genotype, treatment, or gender (**Figures 2A–E**). In the PFC, the outcome was similar, aside from enhanced expression of Ntf3 observed in all male mice (two-way ANOVA: genotype *F*_(3,38)_ = 5,98, *p* < 0.01) but not female mice (however, the results did not reach statistical significance in w/t and Creb1^TPH2CreERT2^ mice exposed to fluoxetine treatment) (**Figures 3A–E**). Additionally, in the PFC, we noticed enhanced expression of Ngf in only Creb1^TPH2CreERT2^ female single mutants (two-way ANOVA: genotype *F*_(3,40)_ = 3,93, *p* < 0.05) in both fluoxetine-treated (*post hoc*, *p* < 0.05) and non-treated (*post hoc*, *p* < 0.05) animals (**Figures 3B,C**).

### Selective Ablation of CREB in Serotonergic Neurons Counteracts the Upregulation of BDNF Evoked by Chronic Fluoxetine Administration

**FIGURE 4:**
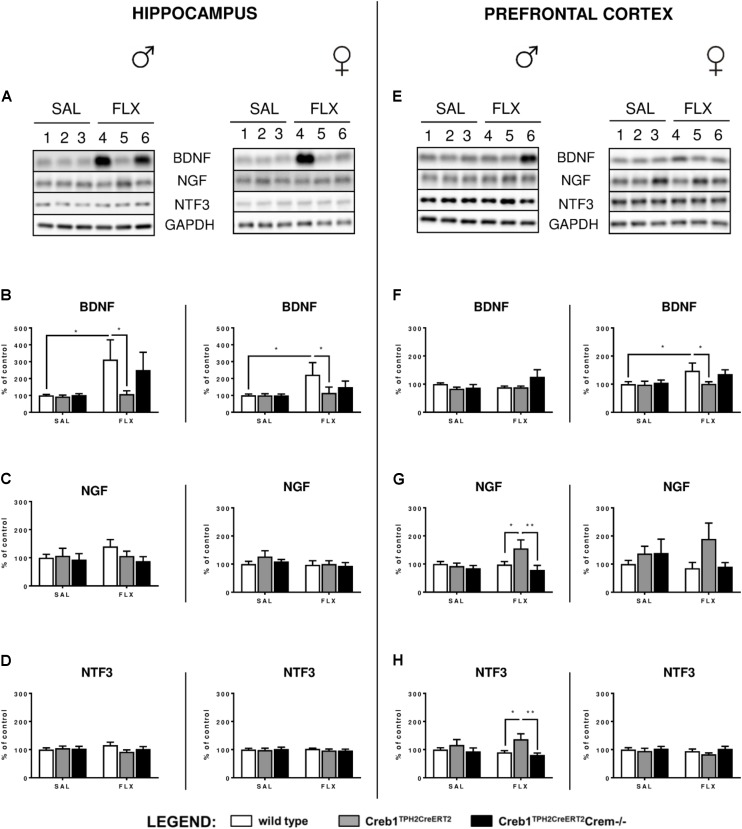
Protein expression of BDNF, NGF, and NTF3 after chronic fluoxetine administration in the hippocampus and PFC of wild-type, Creb1^TPH2CreERT2^ and Creb1^TPH2CreERT2^Crem–/– mice. Chronic fluoxetine treatment (10 mg/kg, ip, 1x daily, 21 days) induced BDNF upregulation in the hippocampus of male and female mice **(B)** and in the PFC of female **(F)** wild-type animals; the effect was abolished in Creb1^TPH2CreERT2^ mice **(B,F)**. Fluoxetine increased NGF **(G)** and NTF3 **(H)** levels in the PFC of male Creb1^TPH2CreERT2^ mutants. Western blot analyses of the effects of fluoxetine administration on the protein levels of **(B,F)** BDNF, **(C,G)** NGF, and **(D,H)** NTF3 in the hippocampus (left panel) and the PFC (right panel) of wild-type, Creb1^TPH2CreERT2^, and Creb1^TPH2CreERT2^Crem–/– mice. **(A,E)** Representative blots of BDNF, NGF, NTF3 and GAPDH in saline (wells 1–3)- and fluoxetine (wells 4–6)-treated wild-type (wells 1, 4), Creb1^TPH2CreERT2^ (wells 2, 5) and Creb1^TPH2CreERT2^Crem–/– (wells 3, 6) mice. Data are presented as the mean ± SEM. ^∗^*p* < 0.05, ^∗∗^*p* < 0.01 vs. saline-injected wild-type mice of the same sex. W/t, wild-type; SAL, saline; FLX, fluoxetine.

Since the analysis of the set of basic genes associated with the neurotrophic theory of depression did not provide any conclusive feedback, the next step was to determine the expression of three neurotrophic factors, BDNF, NGF, and NTF3, on the protein level assessed by Western blot. Indeed, we were able to confirm profoundly enhanced expression of BDNF after 21 days of fluoxetine treatment in the hippocampus of both w/t male (two-way ANOVA, fluoxetine: *F*_(1,36)_ = 4,39, *p* < 0.05; *post hoc* *p* < 0.05) and female (two-way ANOVA, fluoxetine: *F*_(1,34)_ = 4,50, *p* < 0.05; *post hoc* *p* < 0.05) mice (**Figures 4A,B**), an effect that has been previously reported after chronic antidepressant treatment in this brain structure (Nibuya et al., 1995; Vaidya et al., 1999), including selective serotonin reuptake inhibitors (SSRIs) (Baj et al., 2012). Similar effects were observed in the PFC but in only w/t female mice (two-way ANOVA, fluoxetine: *F*_(1,34)_ = 4,48, *p* < 0.05; *post hoc* *p* < 0.05) (**Figures 4E,F**). This observation was counteracted by the mutation introduced into Creb1^TPH2CreERT2^ mice lacking CREB in the serotonergic system (**Figures 4A,B,E,F**). Surprisingly, we noticed a restoration of this effect in double mutants (Creb1^TPH2CreERT2^Crem−/− mice). We were able to visualize these findings by immunofluorescent staining with anti-BDNF antibody performed on coronal slices of the PFC in female mice (**Supplementary Figure S1**). Additionally, in male single mutants (Creb1^TPH2CreERT2^ mice), we found enhanced expression of NGF (two-way ANOVA, genotype: *F*_(2,36)_ = 3,47, *p* < 0.05; *post hoc* *p* < 0.05 vs. w/t + FLX, *p* < 0.01 vs. Creb1^TPH2CreERT2^Crem−/− + FLX) and NTF3 (two- way ANOVA, genotype: *F*_(2,36)_ = 4,92, *p* < 0.05; *post hoc* *p* < 0.05 vs. w/t + FLX, *p* < 0.01 vs. Creb1^TPH2CreERT2^Crem−/− + FLX) evoked by chronic fluoxetine administration in PFC (**Figures 4E,G,H**), but not in the hippocampus (**Figures 4A,C,D**).

### Selective Ablation of CREB in Serotonergic Neurons Does Not Influence the Level and Activity of CREB in the Hippocampus and Prefrontal Cortex

**FIGURE 5:**
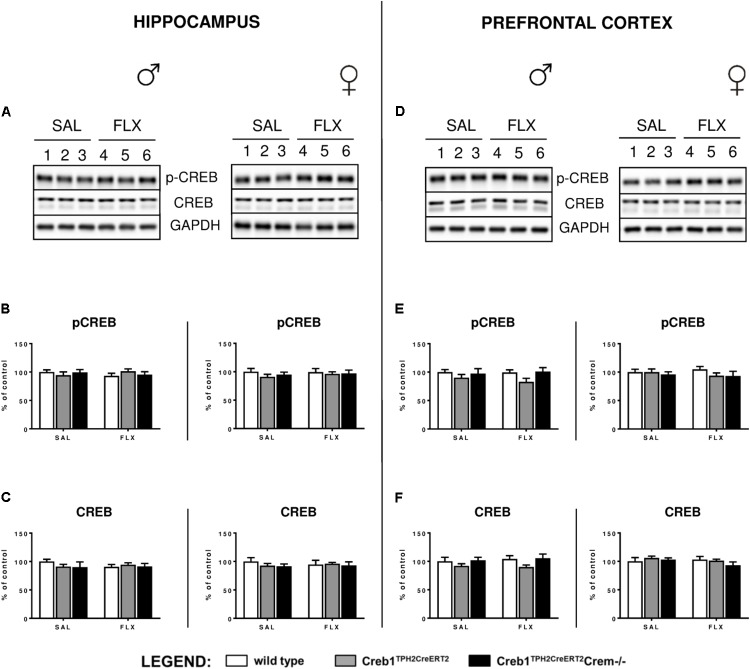
Lack of effect of chronic fluoxetine administration on CREB protein expression and phosphorylation levels in wild-type, Creb1^TPH2Cre^ and Creb1^TPH2CreERT2^ mice. No changes in the protein levels of pCREB and CREB were observed with regard to treatment, genotype, and gender. Western blot analyses of the effects of fluoxetine administration on the protein levels of **(B,E)** phosphorylation at Ser133 CREB (p-CREB) and **(C,F)** total CREB in the hippocampus (left panel) and PFC (right panel) of wild-type, Creb1^TPH2CreERT2^, and Creb1^TPH2CreERT2^Crem–/– mice. **(A,D)** Representative blots of p-CREB, CREB, and GAPDH of saline (wells 1–3)- and fluoxetine (wells 4–6)-treated wild-type (wells 1, 4), Creb1^TPH2CreERT2^ (wells 2, 5) and Creb1^TPH2CreERT2^Crem–/– (wells 3, 6) mice. Data are presented as the mean ± SEM. W/t, wild-type; SAL, saline; FLX, fluoxetine.

Since it is well-known that BDNF expression is regulated by CREB (Finkbeiner et al., 1997), we checked whether the abolition of fluoxetine-induced BDNF overexpression observed in Creb1^TPH2CreERT2^ mice might have also been reflected in the level of CREB functionality. Therefore, we examined the expression of CREB and CREB phosphorylation on Ser-133 by Western blot. However, thorough analysis of all experimental groups did not reveal any changes in CREB and phospho-CREB (pCREB) with regards to genotype, gender and treatment (**Figures 5A–F**).

### The Effects of CREM Deletion on the Expression of Neurotrophic Factors in the Hippocampus and Prefrontal Cortex After Fluoxetine Treatment

**FIGURE 6:**
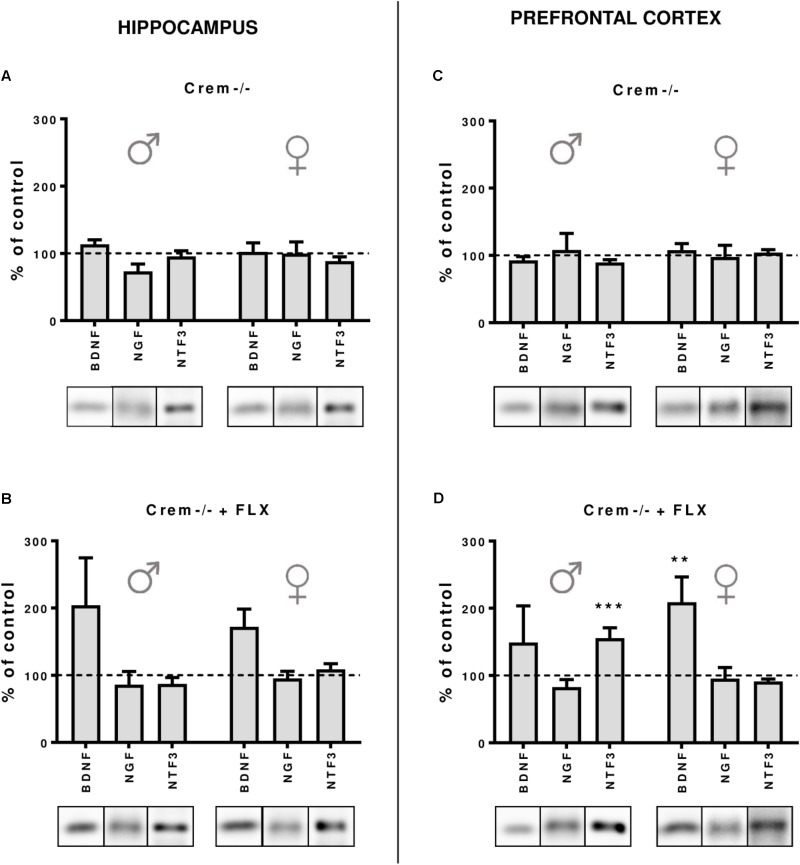
Protein expression of BDNF, NGF and NTF3 after chronic fluoxetine administration in the hippocampus and PFC of wild-type and Crem–/– mice. CREM deletion did not cause any changes in neurotrophin levels in the hippocampus **(A)** or in the PFC **(C)** of male and female mutants. Fluoxetine administration increased BDNF levels in the PFC of females (*p* < 0.01) **(D)**, and a similar tendency was observed in the hippocampus of both Crem–/– males and females. Additionally, fluoxetine increased NTF3 levels in the PFC of Crem–/– males. Western blot analyses of the effects of fluoxetine administration on the protein levels of BDNF, NGF and NTF3 in the hippocampus of **(A)** CREM–/– saline-treated and **(B)** Crem–/– fluoxetine-treated mice and in the PFC of **(C)** Crem–/– saline-treated and **(D)** Crem–/– fluoxetine-treated mice vs. those in wild-type saline animals (dashed line). Representative blots of each group are shown below the corresponding bar. W/t, wild-type; SAL, saline; FLX, fluoxetine. Data are presented as the mean ± SEM. ^∗∗^*p* < 0.01, ^∗∗∗^*p* < 0.001 vs. saline-treated wild-type animals of the same sex.

Finally, to dissect whether the changes in the CREB-dependent regulation of BDNF observed in Creb1^TPH2CreERT2^ mice, but not – at least to the extent reaching statistical significance – in Creb1^TPH2CreERT2^Crem−/− double mutants, were influenced by CREM deficiency, we performed a separate study of BDNF, NGF, and NTF3 protein expression by Western blot that was restricted to w/t and CREB−/− mice. These experiments revealed that indeed the CREM−/− background seems to be responsible for enhanced expression of these proteins observed in Creb1^TPH2CreERT2^Crem−/− after fluoxetine treatment. Namely, in both the hippocampus and PFC of female and male mice, the levels of BDNF were upregulated when compared to those in w/t animals (**Figures 6B–D**); however, only the levels in the PFC of female CREM−/− mice were statistically significant (two-way ANOVA, fluoxetine *F*_(1,21)_ = 8,44, *p* < 0.01; *post hoc* *p* < 0.01 vs. w/t control) (**Figure 6D**). Apart from this effect, only the level of NTF3 in male CREM−/− mice was significantly enhanced related to that in w/t mice (two-way ANOVA, genotype × fluoxetine: *F*_(1,23)_ = 18,90, *p* < 0.001, *post hoc* *p* < 0.001 vs. w/t control) (**Figure 6D**).

It is worth noting that in all non-treated CREM−/− animals, the levels of all investigated neurotrophins were the same as in w/t mice (**Figures 6A–C**), which was concomitant to the data revealing the lack of any changes in BDNF, NGF, and NTF3 w/o fluoxetine treatment in Creb1^TPH2CreERT2^ and Creb1^TPH2CreERT2^Crem−/− mice (**Figures 5A–F**).

## Discussion

The results of this study are a step forward in our research in dissecting the role of CREB in depression and antidepressant treatment. In our study we utilized a novel transgenic mouse model characterized by selective ablation of CREB restricted to chosen neuronal populations, which are important targets for common antidepressant therapies (Rafa-Zablocka et al., 2017).

Here, we show for the first time that BDNF upregulation in the hippocampus or PFC observed after antidepressants targeting the serotonergic system (i.e., fluoxetine) might be dependent on the transcription factor CREB residing not within these particular structures that are targeted by serotonergic projections, but exclusively in serotonergic neurons. This observation may bring a new perspective to the neurotrophic hypothesis of depression, in which the effects of BDNF observed after antidepressants in the hippocampus and other brain structures are thought to be regulated by CREB residing within the same brain structures (Nibuya et al., 1996). In particular, it has been shown that in the hippocampus CREB level is increased after chronic treatment with fluoxetine, which directly augments BDNF expression (Nibuya et al., 1996; Tiraboschi et al., 2004). Since CREB and BDNF both play an important role in neuronal plasticity, both molecules are often regarded as key factors in the pathophysiology of depression and as targets of antidepressant drugs (Nair and Vaidya, 2006). Furthermore, BDNF and serotonergic system are in tight interconnection in the brain regulating the neuronal plasticity, response to stress stimuli and antidepressants efficacy (Mattson et al., 2004; Martinowich and Lu, 2008; Homberg et al., 2014). However due to the heterogeneity of serotonergic transmission the direct interdependence is not so easy to define (Homberg et al., 2014). It has been assured, that chronic administration of BDNF (both *in vitro* and *in vivo*) increases the serotonergic transmission (Celada et al., 1996; Siuciak et al., 1996; Deltheil et al., 2008). Concomitantly, reduced BDNF level observed in BDNF heterozygous KO mice results in the alteration of 5-HT receptor expression associated with decreased serotonin transmission (Lyons et al., 1999). In parallel, there are also existing evidences for regulating BDNF levels by evoking changes in serotonin transmission. Namely, serotonin application *in vitro* (Galter and Unsicker, 2000), and pharmacological stimulation of 5-HT2A receptor *in vivo* by 4-iodo-2,5-dimethoxyphenylisopropylamine (DOI) influenced BDNF expression (Vaidya et al., 1997). These observations were supported by studies performed on knockout animals, showing reduced expression of BDNF in SERT KO rats (Molteni et al., 2010). However, it has to be mentioned, that this regulation is not completely understood and the data are inconsistent – in particular, SERT KO mice did not confirm this latter observation, and Tph2 KO mice were surprisingly characterized by elevated levels of hippocampal BDNF (Kronenberg et al., 2016). The gender differences in studying this issue, in particular in transgenic animals, have also been taken into consideration (Chan and Ye, 2017).

In our experimental model, we did not see any changes in CREB mRNA expression, protein expression or phosphorylation level in the hippocampus nor PFC (**Figures 2**, **3**, **5**). Nevertheless, we cannot exclude the role of CREB in these particular brain structures in raising the BDNF level, as it has been shown that Ser133 phosphorylation is not required for CREB-mediated transcription (Briand et al., 2015). However, it seems to be clear that the changes in BDNF observed after chronic fluoxetine treatment depend on CREB in serotonergic neurons, as the mice selectively lacking CREB in these particular neurons (Creb1^TPH2CreERT2^) lost the ability to show enhanced expression of BDNF after drug administration.

The lack of change observed in CREB in w/t mice after fluoxetine administration does not necessarily constitute a denial of existing hypotheses in this topic. First, it is not a general rule that the correlation between mRNA and protein abundance is straightforward (Maier et al., 2009), and existing data on the effect of long-term antidepressant administration on CREB mRNA and protein expression are inconsistent. For example, both fluoxetine (SSRIs) and desipramine (a drug mainly acting on the noradrenergic system, similar in action to selective noradrenaline reuptake inhibitors, NSRI) have been shown to raise mRNA levels encoding CREB in the hippocampus, but an increase in CREB protein in this structure was observed only after fluoxetine administration (Nibuya et al., 1996). Moreover, other authors have not confirmed the effects of fluoxetine but showed that both antidepressants exacerbated the phosphorylation of CREB in the PFC (Tiraboschi et al., 2004; Laifenfeld et al., 2005). Furthermore, treatment with venlafaxine, a dual serotonin and noradrenaline reuptake inhibitor, significantly reduced pCREB in this brain structure without influence on total CREB expression (Rossby et al., 1999). A generally accepted statement is that chronically used antidepressants should contribute to the enhancement of both expression and activity of CREB (Blendy, 2006) mostly confirmed by data obtained from postmortem studies showing elevated CREB levels in patients that had undergone antidepressant therapy (Dowlatshahi et al., 1998) and decreased levels in those who had not undergone treatment (Yamada et al., 2003). Nevertheless, it remains an open question as to how the involvement of CREB is significant in antidepressant mechanisms and whether its activation is necessary for their effectiveness, as the experiments performed on transgenic animals lacking CREB showed rather opposing effects (genetic deletion of CREB unexpectedly contributed to the induction of an antidepressant phenotype) (Pliakas et al., 2001; Conti et al., 2002; Newton et al., 2002). It should be emphasized, however, that these models were subject to significant limitations that could affect animal behavior, developmental changes and, consequently, interpretation of the results as they were based on non-selective removal of the CREB encoding gene from multiple brain structures. Here, we took advantage of a far more advanced genetic tool allowing for selective and specific deletion of CREB in a chosen neuronal population, i.e., serotoninergic cells; therefore, the effects observed here can be confidently associated with CREB located in these neurons only.

In our experiments focused on the PFC, we found that only the females showed enhanced expression of BDNF after chronic treatment with fluoxetine (**Figures 4E,F**). The topic of gender differences in response to antidepressants emerged recently in clinical studies (Keers and Aitchison, 2010) and has also been observed in behavioral studies conducted on animal models with SSRIs, including our previous studies (Bhatnagar et al., 2004; Jones and Lucki, 2005; Chmielarz et al., 2013; Rafa-Zablocka et al., 2017). Moreover, it has also been shown that chronic fluoxetine, applied in the same dosage as in our experimental paradigm (10 mg/kg) with increased BDNF levels in the hippocampus of both sexes, and this effect was not correlated with cell proliferation, as female mice had higher levels of cell proliferation than their male counterparts. The differences in the pharmacokinetics of fluoxetine may contribute to this phenomenon, as females show higher concentrations of the norfluoxetine metabolite than males in both the plasma and brain (Hodes et al., 2010). Norfluoxetine acts as an SSRI similar to fluoxetine, but its increased half-life may lead to prolonged therapeutic coverage in females, which may translate to the higher brain plasticity observed by Hodes et al. (2010). This effect may also be reflected in our studies.

Gender dition of another neurotrophic factor, neurotrophin 3 (NTF3) (**Figures 4E,G,H**). Here, we found upregulation of mRNA expression encoding for NTF3 in the PFC (but not in the hippocampus) in virtually all investigated male groups but not female groups in comparison to control untreated mice, though not always reaching statistical significance (**Figures 3A–E**). These effects were not always correlated in terms of the protein level, as Western blot performed on the protein samples extracted from the PFC showed enhanced NTF3 expression in only single mutants (Creb1^TPH2CreERT2^ mice) after fluoxetine, while this upregulation ceased again in double mutants (Creb1^TPH2CreERT2^Crem−/− mice) receiving this drug (**Figures 4E,H**). Similar effects regarding the pattern of expression of nerve growth factor, NGF, were observed and do not seem to be gender dependent; however, statistical significance was not reached in females (**Figures 4E,G**). Overall, these observations were somewhat surprising, as the regulation of NTF3 and NGF seems to be opposite of that of BDNF in the mice lacking CREB in serotonergic neurons; however, one must take into consideration that the effects on NTF3 and NGF are restricted to the PFC but not to the hippocampus and to male mice but not to female mice. On the other hand, it is known that chronic treatment with fluoxetine itself (without combination with other drugs) does not increase the levels of NTF3 and NGF in the hippocampus or in the PFC (Agostinho et al., 2011); therefore, observed effects on single CREB-deficient mice (Creb1^TPH2CreERT2^) cannot be regarded as an artifact. In fact, opposite regulation of these three neurotrophic factors was reported previously in various experimental studies (Rocamora et al., 1994; Canals et al., 1998) as well as recently in clinical studies (Bilgic et al., 2017).

All the above described effects regarding the CREB-mediated response of BDNF after fluoxetine treatment were observed predominantly in single mutant mice (Creb1^TPH2CreERT2^ mice), while in the double mutants (Creb1^TPH2CreERT2^Crem−/− mice) also lacking cyclic AMP response element modulator (CREM), these effects were attenuated or even showed a tendency to be reversed (**Figures 4A,B,E,F** and **Table 1**). We included the latter cohort of mice in all the analyses due to the known compensatory properties of CREM in the absence of CREB (Hummler et al., 1994; Mantamadiotis et al., 2002). CREM belongs to the CREB/CREM/ATF family of transcription factors that bind to cAMP response elements (CREs) of cAMP-responsive genes (De Cesare and Sassone-Corsi, 2000).

**Table 1:** Expression of mRNA encoding for BDNF and BDNF protein in w/t, Creb1^TPH2CreERT2^ and Creb1^TPH2CreERT2^Crem−/− mice after fluoxetine treatment.

			Hippocampus		Prefrontal cortex	
Sex	Treatment	Genotype	BDNF mRNA	BDNF protein	BDNF mRNA	BDNF protein
MALE	+SAL	Creb1^TPH2CreERT2^	–	–	–	–
		Creb1^TPH2CreERT2^Crem−/−	–	–	–	–
	+FLX	Wild type	–	↑	–	–
		Creb1^TPH2CreERT2^	–	–	–	–
		Creb1^TPH2CreERT2^Crem−/−	–	↑ns	–	↑ns
FEMALE	+SAL	Creb1^TPH2CreERT2^	–	–	–	–
		Creb1^TPH2CreERT2^Crem−/−	–	–	–	–
	+FLX	Wild type	–	↑	–	↑
		Creb1^TPH2CreERT2^	–	–	–	–
		Creb1^TPH2CreERT2^Crem−/−	–	↑ns	–	↑ns

*Arrows represent significant changes in BDNF expression vs. saline treated w/t mice. n/s, not significant; SAL, saline; FLX, fluoxetine.*

Therefore, we wanted to make sure that CREM had no possibility of compensating for CREB in our model. In general, one should rather expect that the compensatory effect of CREM will prevent to observe any changes evoked by single CREB deletion. Apparently, it was not the case in this study, where the effects of chronic fluoxetine treatment on BDNF expression were counteracted already by single CREB removal from 5-HT neurons, and additional CREM KO background surprisingly seems to revert these changes. It is hard to explain why the lack of both CREB and CREM seems to be less harmful considering the effects of fluoxetine in our study. However, it must be mentioned that since the CREM negative background is not specific to the serotonergic neurons (Creb1^TPH2CreERT2^ mutants were inbred into CREM KO mice) and this mutation is constitutive, this may have an unexpected impact on developmental stages evoking other compensatory mechanisms and so-called off-target effects that are often observed in non-spatiotemporal, classic KO animals (El-Brolosy and Stainier, 2017).

To exploit this topic in depth, we decided to compare all CREM lacking mice with/without fluoxetine treatment in order to determine whether the observed effects diminishing the role of CREB ablation in Creb1^TPH2CreERT2^Crem−/− mice were truly related to the introduced CREM deletion. Indeed, this experiment confirmed the initial hypothesis, as all the CREM-deficient mice unveiled slightly enhanced expression of BDNF after fluoxetine irrespective of investigated structure or gender (however, it reached significance in only the PFC of females), while such effect was not observed in animals that did not receive the drug (**Figures 6A–D**). CREM KO mice are known to have impaired spermatogenesis processes, making the male CREM−/− mice infertile (Blendy et al., 1996), and they are also characterized by emotional and locomotor disturbances (Maldonado et al., 1999). To the best of our knowledge, there have been no studies that have considered the effects of antidepressants on CREM-deficient mice. Nevertheless, CREM is known to play an important function in a variety of physiological responses including cardiac function (Muller et al., 2003). Although it remains speculation, malfunction of cardiac rhythm may put these mice in a more vulnerable state for hypertension induced by fluoxetine, a side effect associated with SSRIs known from animal studies (Hong et al., 2017) and reported in clinics (Jayarajan et al., 2014). If so, this may have a direct influence on BDNF levels reported to be increased in response to hypertensive stimuli (Vermehren-Schmaedick et al., 2013; Erdos et al., 2015).

Surprisingly, we also found that male CREM−/− mice were characterized by significant increases in NTF3 to an extent similar to those observed in single Creb1^TPH2CreERT2^ male mutants. Here, in contrast to the effects observed in BDNF, it seems that both single deletions of either CREB or CREM were sufficient to unleash enhanced expression of NTF3 after fluoxetine, while this effect was not observed in double mutants lacking both CREB and CREM. One may only speculate that compensatory processes evoked in both single mutations may lead to this unforeseen effect; however, this phenomenon remains difficult to explain.

Overall, these results summarized in **Table 1** provide further evidence for the important function of CREB in serotonergic neurons in antidepressant drug action through regulation of BDNF. Confirming our initial findings, we revealed a pivotal role for CREB in these neurons observed after acute response to fluoxetine (Rafa-Zablocka et al., 2017). It is rather hard to compare the data obtained in this work after chronic, 21-days paradigm of fluoxetine administration, with the behavioral response of acute, one-dose fluoxetine administration described in previous study. However, indeed one may speculate that some adaptive changes evoked by removal of CREB from 5-HT neurons may influence also the response observed after acute drug administration. In particular, lack of enhancement of BDNF expression in response to chronic fluoxetine administration may be regarded as an indirect proof that the serotonergic pathway, important for SSRI effectiveness, is somewhat impaired which may be reflected on behavioral level as well. However, it remains speculative as we do not have yet any data regarding the 5-HT neurons functioning in our model.

We believe that our results may provide a valuable contribution to the discussion of the role of CREB in antidepressant drug action and depression, noting that CREB-dependent regulation of neurotrophin responses observed after some antidepressants can be associated not only with the neuronal structures traditionally regarded as important key players in depression pathology but also directly in the neurotransmitter targets of antidepressant therapies.

## Author Contributions

GK designed the study. KR-Z performed the Western blot and RT-qPCR assays. KR-Z and GK performed drug injections, tissue dissections, analyzed the data, and wrote the paper. MB and GK performed immunohistochemistry. MB maintained the transgenic mouse colony and performed genotyping. IN supervised the study.

## Conflict of Interest Statement

The authors declare that the research was conducted in the absence of any commercial or financial relationships that could be construed as a potential conflict of interest.

## Supplementary Material

FIGURE S1 Expression of BDNF in female w/t, Creb1^TPH2CreERT2^, and Creb1^TPH2CreERT2^Crem−/− mice after fluoxetine treatment as visualized by immunohistochemistry. Immunofluorescent staining revealed no visible differences between non-treated w/t and mutant mice **(A–C)**; on the other hand enhanced expression of BDNF-positive cells was noted in w/t mice after fluoxetine **(D)**, an effect no longer observed in transgenic animals **(E,F)**. Immunofluorescent staining performed on paraffin-embedded 7 μM microtome cortical slices of female w/t, Creb1^TPH2CreERT2^ and Creb1^TPH2CreERT2^Crem−/− mice with anti-BDNF antibody (green). -FLX, saline treatment; +FLX, fluoxetine treatment. Scale bar for all pictures: 25 μm.
