# Supplementary figures and images for: Selective Depletion of CREB in Serotonergic Neurons Affects the Upregulation of Brain-Derived Neurotrophic Factor Evoked by Chronic Fluoxetine Treatment
Source: Front Neurosci. 2018 Sep 20;12:637. doi: 10.3389/fnins.2018.00637 (PMC6158386; doi:10.3389/fnins.2018.00637)

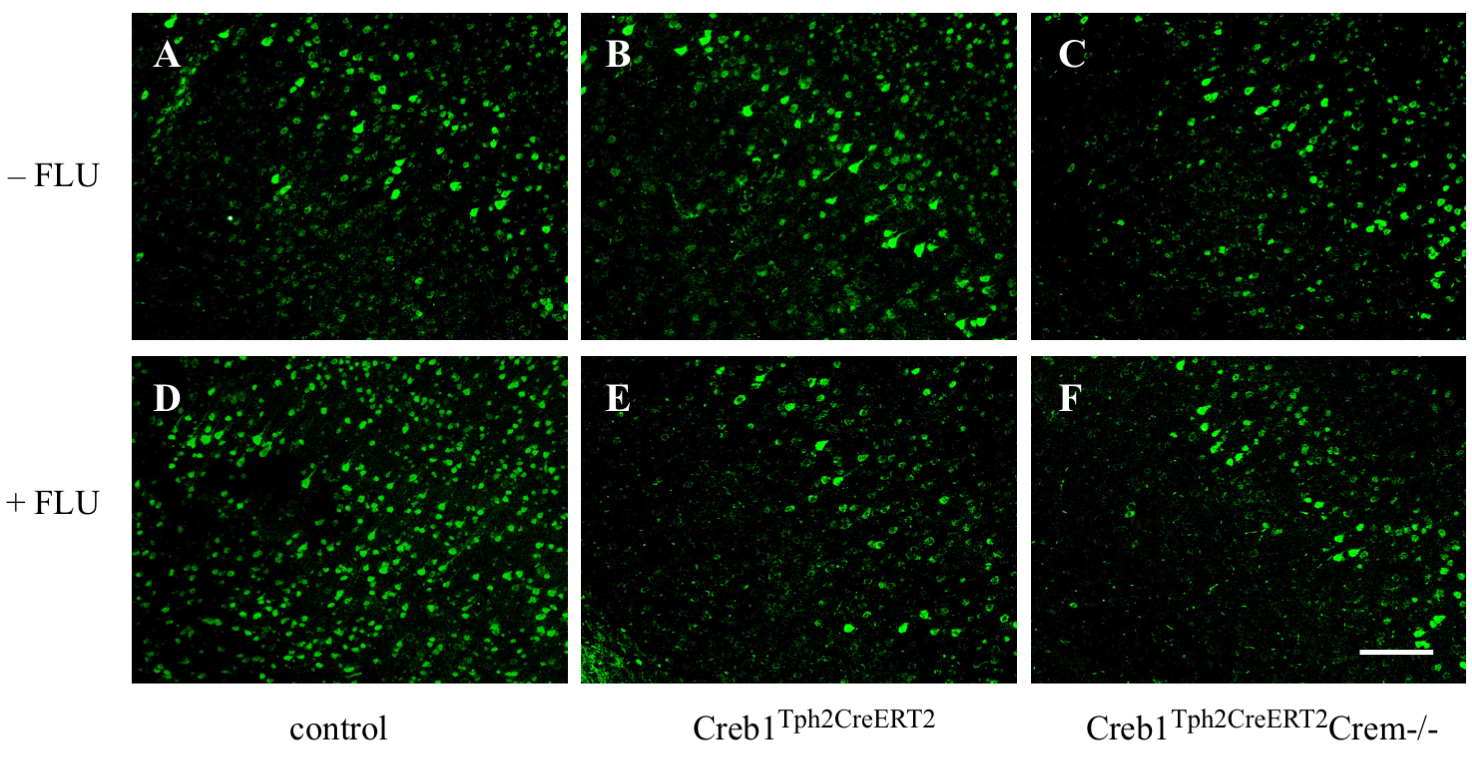

Supplement: FIGURE S1 — Expression of BDNF in female w/t, Creb1TPH2CreERT2, and Creb1TPH2CreERT2Crem−/− mice after fluoxetine treatment as visualized by immunohistochemistry. Immunofluorescent staining revealed no visible differences between non-treated w/t and mutant mice (A–C); on the other hand enhanced expression of BDNF-positive cells was noted in w/t mice after fluoxetine (D), an effect no longer observed in transgenic animals (E,F). Immunofluorescent staining performed on paraffin-embedded 7 μM microtome cortical slices of female w/t, Creb1TPH2CreERT2 and Creb1TPH2CreERT2Crem−/− mice with anti-BDNF antibody (green). -FLX, saline treatment; +FLX, fluoxetine treatment. Scale bar for all pictures: 25 μm. [file Image_1.TIF]
